# Clinical genetics and genomic medicine in Qatar

**DOI:** 10.1002/mgg3.474

**Published:** 2018-09-27

**Authors:** Nader Al‐Dewik, Mariam Al‐Mureikhi, Noora Shahbeck, Rehab Ali, Fatma Al‐Mesaifri, Laila Mahmoud, Amna Othman, Mariam AlMulla, Reem Al Sulaiman, Sara Musa, Ghassan Abdoh, Karen El‐Akouri, Benjamin D. Solomon, Tawfeg Ben‐Omran

**Affiliations:** ^1^ Section of Clinical and Metabolic Genetics Department of Pediatrics Hamad Medical Corporation Doha Qatar; ^2^ Department of Pediatrics Newborn Screening Unit Hamad Medical Corporation Doha Qatar; ^3^ GeneDx Gaithersburg MD; ^4^ Weill Cornell Medical College Doha Qatar; ^5^ Sidra Medicine Doha Qatar

## Abstract

Clinical genetics and genomic medicine in Qatar.

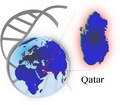

## 1 GEOGRAPHY AND HISTORY OF QATAR

**Figure 1:**
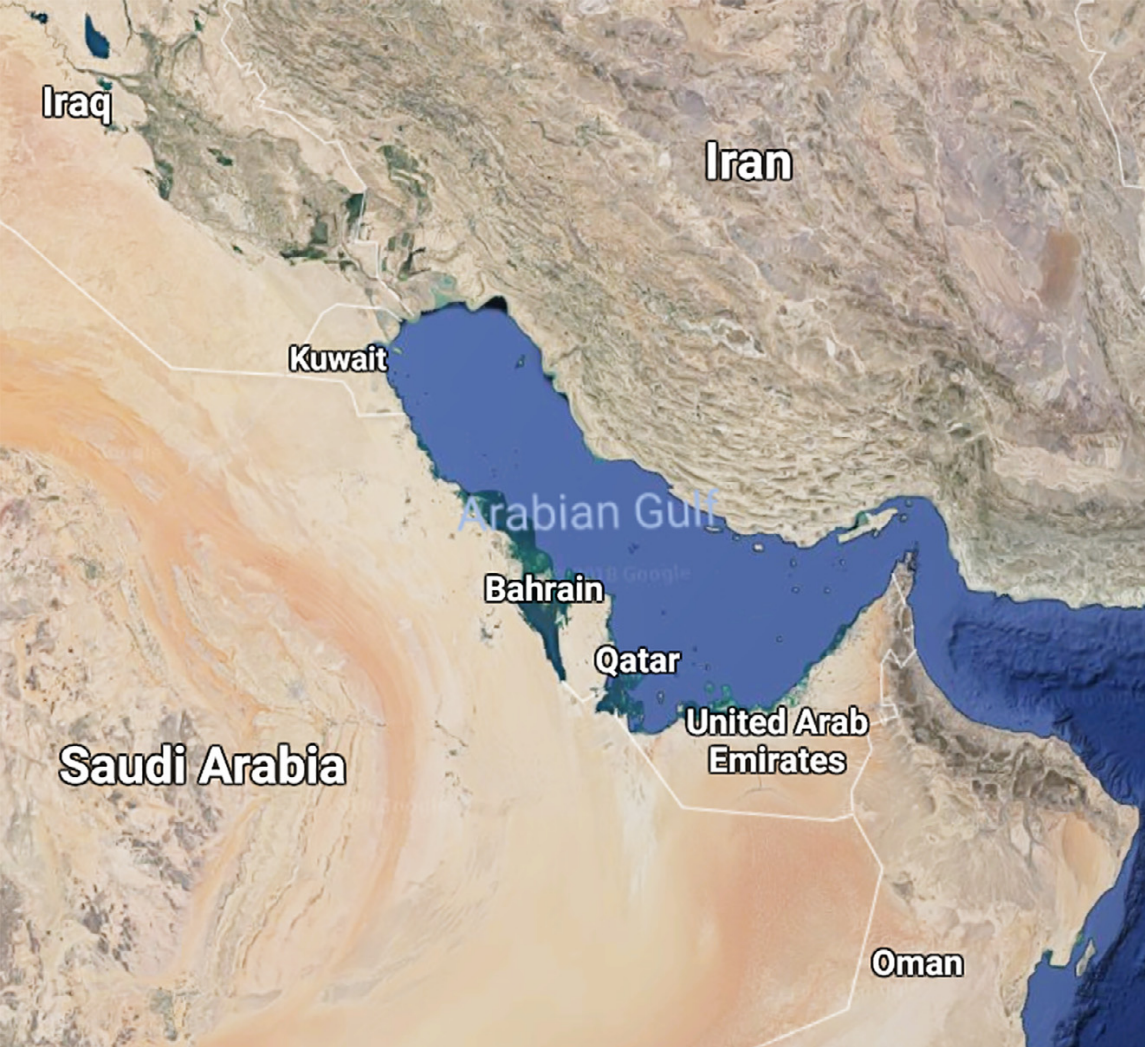
Geography of Qatar and the surrounding region

The State of Qatar (referred to as “Qatar” in this article) is a sovereign Middle Eastern nation located on the northeastern coast of the Arabian Peninsula. Qatar borders Saudi Arabia to the South and is otherwise surrounded by the Arabian/Persian Gulf, with maritime boundaries shared with Bahrain, Iran, and the United Arab Emirates (Figure 1). Qatar's total land area is about 11.5 thousand square kilometers and has a population of approximately 2.7 million, most of which lives in and around the capital, Doha.

Qatar has a rich and fascinating history and has been occupied by humans for approximately 50,000 years. Many different populations have inhabited the area, as its location led to an influx of Arab tribes from the surrounding region, especially the Nejd desert to the West. Islam began to flourish in what is now Qatar in the seventh century CE, and the area was important in the spread of Islam. The Portuguese Empire took control of the region early in the sixteenth century, but was soon overthrown by the Ottoman Empire, which subsequently ruled Qatar for several hundred years. Qatar became a British protectorate in 1916 and declared independence on 3 September 1971.

Currently, Qatar is a highly developed and influential country in the region. Its economic development has led to major advances in all sectors of the country. The healthcare sector in Qatar is distinguished by its numerous international accreditations, providing the highest standards of care and offering state‐of‐the‐art services both generally and as directly relates to the practice of genetics and genomics in clinical and research settings.

## 2 QATAR HEALTH STRATEGY AND NATIONAL VISION

Qatar's National Health Strategy (NHS) 2011‐2022 was launched by Her Highness Sheikha Moza bint Nasser in April 2011. The NHS consists of two phases: (a) National Health Strategy 2011–2016, which has the structure for current development such as growth of investment in new healthcare facilities, services and healthcare infrastructure to meet the needs of the nation's fast‐growing population (Supreme Council of Health, 2013), and (b) the National Health Strategy 2018–2022 for the next period of growth under the theme “Our Health Our Future”, which reflects a shift in thinking by focusing on the seven priority population groups: healthy children and adolescents, healthy women leading to healthy pregnancies, healthy and safe employees, mental health and well‐being, improved health for people with multiple chronic conditions, health and well‐being for people with special needs, and healthy aging.

The new strategy identified 19 national goals against which progress will be measured, including specific goals to promote physical activity among adolescents, to initiate workplace welfare programs, and to offer improved access to mental health services.

The strategy focuses on five areas of the health system to ensure an integrated system for high‐quality health care and services. Its targets by 2022 include reducing the overall rate of avoidable death by 5% and reducing the rate of hospitalization for preventable primary care cases by 15% (Ministry of Public Health, 2018).

This will introduce a new and more integrated approach to Qatar's health challenges and care. Overall, the goal is to shift the focus from diseases among individuals to the health of populations and from acute care to primary care, from health cost as a financial load to a future investment and from patients receiving medical advice to an individual controlling their own health. Annually, the implementation of the strategy will be assessed and reviewed regarding the overall health needs of Qatar's population.

Human development is the most important element of Qatar National Vision (QNV) 2030, an ambition plan for Qatar to develop and sustain a healthy population (physically and mentally) by spending 2.2% of the country's GDP on health care. The 2018 budget has allocated QR22.7 billion funds for the health sector, representing 11.2% of the total expenditure in 2018 (General Secretariat For Development Planning, 2008; The Peninsula, 2017).

## 3 THE QATARI POPULATION AND CULTURAL INFLUENCES

The population of Qatar forms a multiethnic community; approximately 600,000 (22%) of the 2.7 million population are native Qataris (largely tribal) (Ministry of Development Planning and Statistics); expatriates make up the remaining portion of the population. This could be attributed to the country's strong economic performance, fueling and contracting services staff, as well as expatriate professionals returning to the country.

The native Qatari population is characterized by a high consanguinity rate. According to one analysis conducted over a decade ago, more than 54% of marriages were between first or second cousins (Bener & Hussain, 2006), though this rate may be increasing. A high rate of consanguinity is similarly seen in numerous expatriate communities in Qatar. Together with consanguinity, the relatively large family size plays a role in the high prevalence of autosomal recessive conditions in the population of Qatar.

A more recent analysis (2012–2013) was conducted at two medical centers in Qatar: the Clinical and Metabolic Genetic Division of Hamad Medical Corporation (HMC) and the Shafallah Rehabilitation Center (SRC). In that study, consanguineous marriages were observed in 397 (67.7%) of 599 Qatari families seen. As anticipated, this was observed to confer a significantly higher incidence of autosomal recessive disorders compared to nonconsanguineous marriages ([total cohort HMC and SRC; (odds ratio = 1.72; 95% CI: 1.10, 2.71; *p* = 0.02)] [HMC; (odds ratio = 2.98; 95% CI: 1.37, 6.09; *p* = 0.005)]) (unpublished data). Further, autosomal recessive disorders were found to be more likely predicted by couples involving paternal first cousin (OR: 3.70; 95% CI: 1.44–9.52; *p* = 0.007), double first cousin (OR: 6.17; 95% CI: 1.25–30.32; *p* = 0.025) and first cousin (OR: 4.44; 95% CI: 1.49–13.23; *p* = 0.007) (unpublished data).

Additionally, the high rate of consanguinity favors the coexistence of multiple distinct autosomal recessive disorders within single families. Even more complex families have been diagnosed with a combination of autosomal recessive disorders and other single‐gene disorders with different inheritance patterns. The recent advances in genomic sequencing technologies have helped identify patients affected by more than one genetic condition, such as LIG4 syndrome and urofacial syndrome (Fadda et al., 2016) and cystic fibrosis and apparent mineralocorticoid excess (AME) syndrome (Zahraldin et al., 2015). Clinicians and researchers working in Qatar have to be particularly mindful of the possibility that a patient's overall phenotype may be due to a combination of different, coincidental genetic conditions (versus directly interacting genetic factors) (Retterer et al., 2016). In other words, geneticists are mindful not to “stop” analyses at the identification of one genetic condition when features of a patient's overall phenotype remain unexplained.

As with all modern societies, religious, cultural, and ideological beliefs play a central role in the practice of medicine and clinical genetics. For example, over the past years, prenatal genetic services have become more accepted and requested by Arab families not only to provide reassurance or early diagnosis, but also to inform pregnancy management options. The option of termination of pregnancy is becoming more permissible and accepted not only if the mother's life is endangered (Al Aqeel, 2010; Al‐Aqeel, 2005; Albar, 2002; El‐Hazmi & Ai‐Aqeel, 2006; Hathout, 1992), but also for severe and life‐threatening fetal conditions. Assisted reproductive technologies (ARTs) such as in vitro fertilization (IVF) and preimplantation genetic diagnosis (PGD) may be used when indicated as long as the couple's gametes are used (Teebi, 2010).

Other recent bioethical issues at the heart of heated discussions across the world, such as secondary or incidental findings found through exome/genome analyses, have also been addressed from an Islamic bioethical perspective by Qatar's leadership in conjunction with other genomics leaders. Major recommendations and conclusions of this initiative are summarized and directly quoted as follows: potential recipients of incidental findings should be properly informed; incidental findings that can lead to actionable lifesaving procedures should be disclosed; incidental findings related to (misattributed) paternity should not be disclosed; a one‐size‐fits‐all approach does not work with many incidental findings. Notably, the overall work provides background as well as specific case studies to illustrate situations related to these points (Sadoun et al., 2018).

## 4 GENETICS IN QATAR AND THE ARAB WORLD

Extensive discussions have been published on this topic, both related to specific countries and populations as well as more broadly; see Teebi's textbook on the subject for a comprehensive overview (Teebi, 2010). While the purpose of this review article is not to recapitulate previous works such as that textbook, several themes emerge that are worth noting.

First, Qatar and other countries in the region have served as key areas for the discovery of novel genetic causes of human disease (Scott et al., 2016). In many cases, these causes were first identified in this region through clinically oriented research endeavors and then extrapolated to other parts of the world. That is, after identifying individuals with causative genetic changes in the population of Qatar and the region, individuals in other parts of the world with the same phenotypes were found, tested, and identified to have the same underlying genes involved, though sometimes with compound heterozygosity rather than homozygosity for pathogenic variants.

There are several explanations for this history of discovery, including strong economic resources and the willingness to invest in and use genomic technologies and research, histories of collaborative work with other large and international partners (e.g., Weill Cornell University, USA; University Children's Hospital of Heidelberg, Germany; Boston Children Hospital, USA; Rockefeller University, USA; University of Zurich, Switzerland – see more details below), and cultural practices that enable the investigations of the causes of conditions such as via homozygosity mapping. As mentioned (Teebi, 2010), the genetic causes of hundreds of individual conditions were first described in these populations, and new explanations for genetic diseases are continually being reported and published. Importantly, these studies have lent themselves to translational work that have helped dissect the underlying biological causes of disease. A few of many recent examples include *TBC1D23* (OMIM 617687) and pontocerebellar hypoplasia (Marin‐Valencia et al., 2017); *SLC7A5* (OMIM 600182) and autism spectrum disorder (Tarlungeanu et al., 2016); *TMTC3* (OMIM 617218) and cobblestone lissencephaly (Jerber et al., 2016); *MBOAT7* (OMIM 606048) and autism with seizures and intellectual disability (Johansen et al., 2016). In addition to gene/condition‐specific work, larger studies that incorporate statistical analyses of the underlying genomic architecture of the population have also yielded novel gene discovery techniques (Scott et al., 2016).

Second, other studies focusing on patients and genomic data from Qatar continue to address a wide spectrum of other hypotheses and areas. These include using the population to explore the effects (or lack thereof) of “knock‐out” alleles in humans (Maddirevula et al., 2018), as well as defining well‐known conditions in the Qatari populace. Recently, the importance of ancestry‐specific descriptions has been recognized and emphasized. That is, even supposedly well‐defined conditions have primarily (or sometimes only) been described in populations of Western European descent, and genetic and nongenetic factors, including patterns of clinical care, modulate and influence the ultimate phenotype and outcomes (Muenke, Adeyemo, & Kruszka, 2016). Knowledge of clinical similarities and differences is especially important for practitioners and researchers caring for diverse sets of patients. Recent examples of conditions with studies involving patients from Qatar include Aicardi‐Goutières syndrome (Al Mutairi et al., 2018) and classic homocystinuria (El Bashir, Dekair, Mahmoud, & Ben‐Omran, 2015).

Third, related to the factors described above, as well as due to having available resources (and the mindset) to adopt newer clinical diagnostic practices early, Qatar has been able to achieve high diagnostic rates through broad application of clinical exome sequencing (CES). Early analyses (2012–2014) of 149 probands undergoing CES identified a causative explanation in 89 (60%) of the studied probands (Yavarna et al., 2015). More recent analyses of a larger cohort of >500 patients show a diagnostic yield of >48% (Al‐Dewik et al., 2017). This still high but slightly lower yield may involve factors such as evolving differences in variant classification techniques and guidelines as well as underlying cohort differences and practices in terms of which patients receive CES and when.

Finally, though somewhat separately, prevalences and characteristics of certain types of congenital anomalies have been studied specifically in the Qatari population. For example, congenital cardiovascular malformations (1984–1994) were reported as having an incidence of ~12:1,000 live births (Robida, Folger, & Hajar, 1997). From another study on congenital brain malformations (1986–1989), the incidence of hydrocephalus was 157:100,000 live births and of meningomyelocele was 41:100,000 (Nogueira, 1992).

## 5 HEALTHCARE SERVICES AND CENTERS OFFERING GENETIC SERVICES IN QATAR

Different healthcare services are delivered through the country by private, private/government, and public providers, who organize the most of healthcare activity. Hamad Medical Corporation (HMC) is the main public healthcare provider in the State of Qatar. Hamad Medical Corporation (HMC) has also been the main provider of clinical and metabolic genetic services for the population of Qatar since 2002. HMC includes 10 hospitals (7 specialty hospitals and 3 community hospitals) as well as ambulance, home, and residential care services (Table 1).

**Table 1:** Hospitals in Qatar operated by Hamad Medical Corporation (HMC)

Year of establishment	Hospital
1957	Rumillah Hospital
1982	Hamad General Hospital
1988	Women's Hospital
2004	National Center for Cancer Care and Research
2005	Al‐Khour Hospital
2011	Heart Hospital
2012	Cuban Hospital; Al‐Wakra Hospital
2016	Communicable Disease Center
2017	Women Wellness and Research Center; Ambulatory Care Center; Qatar Rehabilitation institute

HMC's Clinical and Metabolic Genetics (C&MG) Division is the only national referral center for genetic diseases in Qatar. This division provides state‐of‐the‐art medical care for patients (both pediatric and adult) and families affected by a variety of inherited or genetic disorders. Clinical services include clinical genetics and dysmorphology, inherited metabolic disorders, prenatal genetics, and follow‐up and care for positive cases discovered through the National Expanded Metabolic Newborn Screening and National Premarital Genetic Counseling and Screening programs (see details below). Alongside traditional medical care, the division provides genetic counseling for general genetics, prenatal and Preimplantation Genetic Diagnosis (PGD) services and receives patients from neighboring countries. In 2016, the Clinical and Metabolic Genetics service saw over 11,865 cases in outpatients clinics (in addition to inpatient services) for various known or suspected genetic and metabolic disorders.

**Figure 2:**
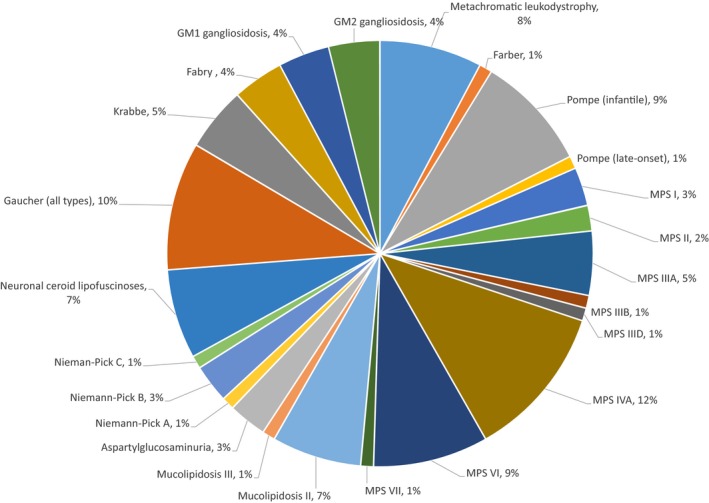
Proportion of lysosomal storage disorders in Qatar of a total 107 patients

As necessitated by the patient base, the division is especially active in the diagnosis and management of inborn errors of metabolism (e.g., enzyme replacement therapy for patients with lysosomal storage disorders) and neuromuscular disorders (Figure 2). As described, the high consanguinity rate, together with large family size and rapid population growth in the population, has led to a high relative frequency of metabolic and genetic diseases. For example, classical homocystinuria is highly prevalent in Qatar (the highest known incidence in the world, ~1:1800). As detailed below, for the overall conditions screened, dozens of positive patients are identified each year through Qatar's National Expanded Metabolic Newborn Screening program.

Separate from work that may be performed by reference laboratories, various common and rare chromosomal abnormalities are diagnosed “in‐house” at the HMC cytogenetics laboratory, with the number and rate of diagnosis logically increasing after the introduction of CGH arrays (Nair, Obeid, & Tadmouri, 2012). For example, the most common diagnosed disorder is trisomy 21 (98.3%), which occurs at an incidence of 1:513 live births in Qatar, partly attributed to advanced maternal age (median = 36 years) (Teebi, 2010).

Conditions diagnosed and treated include both rare and common genetic conditions, including genetic disorders that are more common in the country of Qatar than other parts of the world, as well as conditions that appear to occur at approximately the same rate across the globe, such as syndromic forms of craniosynostosis, Marfan syndrome, X‐linked recessive hypophosphatemic rickets, and many others (Teebi, 2010). As expected, all major inheritance patterns have been observed; for a fuller list of conditions, see (Teebi, 2010). These clinical genetic services continue to serve many thousands of families from the region and have provided clinical and molecular diagnoses to many patients.

As mentioned, this approach has allowed the identification of many novel conditions (i.e., due to new candidate genes) in this population as well as observations about conditions that are more frequent such as due to the increased frequency of certain alleles related to autosomal recessive conditions (Table 2). These observations have also informed Qatari screening programs (see section below). In addition to and building upon its clinical services, the Qatar Genome Programme (QGP) was announced in 2013 by her Highness Sheika Moza Bint Nasser, Chairperson of the Qatar Foundation. The QGP was designed within the framework of a comprehensive national plan and is based on seven building blocks: the National Biobank; National Genomics Infrastructure; National Genomics Research Partnerships; National Genome Data Network; qualified local human capacity; policies regulating genomics research in precision medicine; integration of genomics in the clinical setting.

**Table 2:** Examples of conditions observed more commonly in patients seen by Hamad Medical Corporation clinicians

Condition	Comments	Reference(s), where available
Arterial tortuosity syndrome	Described in multiple members in several families due to the NM_030777.3(SLC2A10):c.243C>G (p.Ser81Arg) variant (OMIM 606145)	Abdul Wahab et al. (2003) and Faiyaz‐Ul‐Haque et al. (2008)
Cystic fibrosis	The variant NM_000492.3(CFTR):c.3700A>G (p.Ile1234Val) (OMIM 219700) is a high‐frequency founder variant in the tribal Qatari population and results in a mild‐to‐moderate phenotype	Abdul Wahab, Al Thani, Dawod, Kambouris, and Al Hamed (2001)
Dihydrolipoamide dehydrogenase (DLD) deficiency	Multiple Qatari families have been identified as affected with the NM_000108.4(DLD):c.685G>T (p.Gly229Cys) variant (OMIM 238331)	
Epidermolysis bullosa, junctional type	Multiple members of a Bedouin family have been described with the NM_000227.4(LAMA3):c.3609 + 1G>A variant (OMIM 600805)	Teebi (2010)
Hemoglobinopathies	Multiple pathogenic variants are observed at higher frequencies in the population of Qatar, including variants related to different hemoglobinopathies (e.g., ß‐thalassemia, sickle cell anemia)	Al‐Obaidli et al. (2007)
Homocystinuria due to cystathionine beta‐synthase deficiency	The highest global incidence in the world is reported in Qatar (1:1800); 2% of the population is estimated to carry the NM_000071.2(CBS):c.1006C>T (p.Arg336Cys) variant (OMIM 613381)	Al‐Dewik, Ali et al. (2017), Al‐Dewik, Mohd et al. (2017), El‐Said et al. (2006), Gan‐Schreier et al. (2010) and Zschocke et al. (2009)
Mitochondrial calcium uniporter	Described in multiple members in several families due to the NM_006077.3(MICU1):c.553C>T (p.Gln185Ter) variant (OMIM 605084)	(Musa et al., 2018)
Nonsyndromic microphthalmia/anophthalmia	Described in multiple members in several families due to the NM_182894.2(VSX2):c.599G>C (p.Arg200Pro) variant (OMIM 142993)	Faiyaz‐Ul‐Haque et al. (2007) and Teebi (2010)
Spinal muscular atrophy (SMA)	There are currently 23 Qatari patients living with SMA, of which 17 patients have SMA type 1; 2 patients have SMA type 2; and 4 patients have SMA type 3. Out of these 23 patients, 8 patients were diagnosed in 2017 and 2018 (OMIM 600354)	
Woodhouse–Sakati syndrome	Multiple members of highly consanguineous families from Qatar have been described with the NM_025000.3(DCAF17):c.436delC (p.Ala147Hisfs) variant (OMIM 612515*)*	Ben‐Omran et al. (2011)
Van Den Ende–Gupta syndrome (VDEGS)	Multiple members of an extended Bedouin tribe have been described with the NM_153334.6(SCARF2):c.773G>A (p.Cys258Tyr) variant (OMIM 613619)	Anastasio et al. (2010)

Sidra Medicine has been established in 2017 as a high‐tech facility that provides world‐class patient care in all subspecialties, including genetic and genomic medicine.

## 6 NATIONAL GENETIC SCREENING PROGRAMS: NEWBORN SCREENING AND PREMARITAL SCREENING

Qatar was one of the first Arab countries to establish a national expanded newborn screening (NBS) program, which was implemented in December 2003 (Table 3). This NBS program has been conducted through a partnership with University Children's Hospital of Heidelberg, Germany. From December 2003–July 2006, 25,214 neonates in the State of Qatar were investigated for inborn errors of metabolism and endocrine disorders, revealing an incidence of these conditions (26 disorders) of 1:1,327, compared to 1:2,517 in Germany. The higher incidence in Qatar primarily relates to inborn errors of metabolism; congenital hypothyroidism had approximately the same incidence as observed in Germany (Lindner et al., 2007). Importantly, analyses of this NBS implementation showed that, for every 1 euro spent on this NBS program, over 25 euros were saved in healthcare and social costs.

**Table 3:** Conditions included in newborn screening in Qatar

Disorder	Group
Congenital hypothyroidism	Endocrinopathies
Congenital adrenal hyperplasia	
PKU, HPA, BS	Aminoacidopathies and urea cycle disorders
MSUD	
HCY	
Tyrosinemia type I	
Citrullinemia	
Argininosuccinic aciduria	
Methylmalonic aciduria (Cbl disorders)	Organic acidurias
Propionic aciduria	
Glutaric aciduria type I	
Isovaleric aciduria	
3‐Methylcrotonylglycinuria	
MAD	
MCAD deficiency	Fatty acid oxidation disorders, carnitine cycle defects and disorders of ketogenesis
VLCAD deficiency	
LCHAD/mTFP deficiency	
Carnitine transporter deficiency	
CPT‐I, CPT‐II	
HMG‐CoA lyase deficiency	
Ketothiolase deficiencies	
Classical galactosemia	Others
Biotinidase deficiency	

Note

PKU: phenylketonuria; HPA: benign hyperphenylalaninemia; BS: defects of biopterin cofactor biosynthesis; MSUD: maple syrup disease; HCY: homocystinuria (due to cystathionine beta‐synthase deficiency); MAD: multiple acyl‐CoA dehydrogenase deficiency; IBDH: isobutyryl‐CoA dehydrogenase deficiency; MCAD: medium‐chain acyl‐CoA dehydrogenase deficiency; VLCAD: very long‐chain acyl‐CoA dehydrogenase deficiency; LCHAD: long‐chain 3‐hydroxy acyl‐CoA dehydrogenase deficiency; mTFP: trifunctional protein deficiency; SCAD: short‐chain acyl‐CoA dehydrogenase deficiency; CPT‐I: carnitine palmitoyltransferase I deficiency; CPT‐II: carnitine palmitoyltransferase II deficiency; HMG‐CoA lyase deficiency: 3‐hydroxy 3‐methyl glutaric aciduria.

In a follow‐up study conducted from December 2003–September 2015, of 208,042 newborns born in Qatar, 296 neonates were positive for NBS conditions (185 patients with metabolic and 111 with endocrine disorders). Estimated incidences of metabolic and endocrine disorders were 1:711 and 1:1,671, respectively. Of the NBS‐positive infants, 91 had congenital hypothyroidism, 20 had congenital adrenal hyperplasia, 39 had classical homocystinuria, 38 had other aminoacidopathies or urea cycle disorders, 38 had fatty acid oxidation disorders, 30 had organic acidemias, 12 had biotinidase deficiency, and 14 had galactosemia. As with other programs in other countries and regions, these services continue to evolve with increasing knowledge of high‐risk conditions with available interventions as well as related to technological advancements (Gramer et al., 2017).

In 2009, the National Premarital Genetic Screening program was launched as a mandatory step for all couples planning for marriage in Qatar. The screening is offered by designated primary healthcare centers and selected private hospitals for hemoglobinopathies (thalassemias, sickle cell disease); classical homocystinuria; cystic fibrosis; and optionally spinal muscular atrophy (SMA).

The above‐mentioned conditions were selected for screening because of high carrier frequencies in the population of Qatar. Screening for hemoglobinopathies is performed by complete blood count (CBC) and hemoglobin electrophoresis, followed by molecular genetic testing as indicated. Screening for classical homocystinuria and cystic fibrosis is primarily offered for known founder mutations (see Table 2). Carrier testing for SMA is performed by targeted deletion/duplication testing to detect the common exon 7 deletion in the *SMN1* gene. A challenge in screening for SMA in Qatar was highlighted after the identification of newborns with SMA born to couples in which one parent was an SMA “silent carrier” with two copies of the *SMN1* gene (OMIM 600354) on one chromosome 5 and a second chromosome 5 with zero copies (also known as “2 + 0” genotype).

## 7 PRENATAL GENETIC PROGRAM

The prenatal genetic service was established in 2013, as part of Clinical and Metabolic Genetic services at HMC, in order to further meet the needs of the population of Qatar. This is a first and still unique and leading service in the region and helps to establish clinical practice guidelines related to prenatal genetic diagnosis for all clinicians involved in prenatal care, including family physicians, obstetricians, and geneticists. There is a demand for this service due to high rate of consanguinity leading to high prevalence of genetic and metabolic diseases in Qatar.

The prenatal genetic team provides comprehensive prenatal genetic services including: case review; risk assessment; discussion of testing options; result disclosure; patient education; formal genetic counseling; discussion of pregnancy management options and postnatal care; planning for further family counseling and testing; psychosocial support. The prenatal genetics team consists of a prenatal geneticist, genetic counselors, and genetic counseling assistants, who work in collaboration with Maternal‐Fetal Medicine (MFM) physicians and other subspecialists to manage high‐risk pregnancies and coordinate postnatal care.

## 8 RESEARCH AND INNOVATION PORTFOLIO

The HMC Clinical & Metabolic Genetics Division successfully built their research culture within their area of focus by carrying out collaborative clinical, translational and basic sciences research projects with local institutes as well as international, well‐recognized centers and universities. Their mission is to further the understanding of the genetic components of inherited diseases pertinent to the population of Qatar and the region, including classical homocystinuria and other autosomal recessive conditions relatively common to this population.

This research is specifically designed to support three major themes:

- Disease gene discovery
- The development of new treatment strategies
- Molecularly based dysmorphologic understanding of genetic syndromes, including in prenatal genetics

Their current research activities include a multidisciplinary approach to identify the underlying genetic factors of metabolic and genetic disorders as well as the effects of founder mutations in the Qatari population for certain high‐priority diseases such as classical homocystinuria and white matter disorders, as well as related clinical trials designed to translate research discoveries and findings into practical applications.

This group has secured substantial funding from the Qatar National Research Fund (QNRF), HMC, and Qatar Innovation Promotion Award (QIPA) (Total funding successfully obtained: $6,106,095 from 2011–2018).

The Clinical & Metabolic Genetics Division has been recognized at the international level for the establishment of three clinical trials to date (for spinal muscular atrophy and mucopolysaccharidosis IVA), which are registered in clinicaltrials.gov (a registry and results database of publicly and privately supported clinical studies of human participants conducted around the world under the U.S. National Institutes of Health), and are currently receiving several feasibility packages to participate in future clinical trials involving interventions for homozygous familial hypercholesterolemia (HoFH) and Pompe disease.

Additionally, the work of the Clinical & Metabolic Genetics Division continues to allow basic and translational collaborations with the international scientific community, including large, historied centers and universities such as McGill University and the Genome Quebec Innovation Center, Canada; Boston Children's Hospital, Boston, USA; The Rockefeller University & New York Genome Center, USA; University Hospital Freiburg, Germany; University of Zurich, Switzerland, as well as locally affiliated universities such as Weill Cornell Medical College, Qatar University, and Shafallah Medical Genetic Center. These and other collaborations have led to high‐impact findings published in peer‐reviewed journals and presented at national and international meetings (for example, Dixon‐Salazar et al., 2012; Jerber et al., 2016; Novarino et al., 2012, 2014; Tarlungeanu et al., 2016). The genetic findings have been adopted and integrated in the Q‐Chip, and several initiatives are in process to develop several multigene panels (Marhaba, 2018).

The team will continue running several basic/translational research projects in their laboratory in collaboration with well‐recognized universities and hospitals. Based on past successful projects, their future research plans include:

- Development of novel therapeutic alternatives, including chaperones and gene therapy approaches for homocystinuria
- Regenerating central nervous system white matter using induced pluripotent stem cells
- Development of genomic newborn screening approach.
- Development of comprehensive gene panels (Qatar Mendeliome assay)
- Clinical and molecular genetics characterizations of metabolic and genetic disorders
- Studying the role of epigenetics and transcription factors including noncoding RNA in genetic and metabolic disorders
- Establishment of metabolic and genetic patients’ cell lines, representing patients’ and biological phenotypes
- Development of innovative techniques/methods for metabolic and genetic patients
- Participation in international clinical trials and PI‐initiated clinical trials for metabolic and genetic patients.

## 9 TRAINING PROGRAMS

HMC has established a three‐year fellowship program in the Clinical Genetics & Metabolic Diseases (with ACGME‐I accreditation), which is designed to provide comprehensive clinical (including metabolic) and research training. The program has also been approved by the Medical Education Department at HMC. To date, this program has successfully graduated three fellows (two of whom are Qatari).

Further, the Clinical Genetics & Metabolic team actively participates in undergraduate and graduate teaching programs in several universities across the country and successfully supervises undergraduate and graduate students to their degrees.

In addition, Hamad Bin Khalifa University (HBKU) launched a Genomics and Precision Medicine (GPM) graduate program in 2017. This is a multidisciplinary graduate program designed to prepare the next generation of professionals and leaders who will help implement the use of precision and personalized medicine in the healthcare system. The GPM offers students advanced knowledge and training in state‐of‐the‐art information gathering and analysis technologies. The goal is to further integrate “omics”—the branch of biology that focuses on molecular level data—with other clinical data, enabling the design and implementation of precision medicine in health care within Qatar and the region. Overall, graduate students are given a unique opportunity to study all key elements of genomics and precision medicine in one innovative, multidisciplinary program.

Recently, Qatar University has established a new graduate program in genetic counseling. The overall objective of the program is to educate and train local masters‐level candidates who can become capable of providing genetic counseling to patients and families in Qatar with known or suspected genetic conditions.

## 10 CONCLUSIONS

Qatar is a fascinating example of good clinical practice of genetics, genomic medicine, and translational research due to the strong resources, investment in genetics and genomics, local expertise, leadership, participation in both small and large studies, and early and widespread adoption of new diagnostic and related technologies. Furthermore, genetic services in Qatar provide state‐of‐the‐art care for the population of Qatar and the region.

## DISCLOSURES

N A‐D, M A‐M, NS, RA, F A‐M, K E‐A, MA, R AS, SM, and T B‐O are part of the described Section of Clinical and Metabolic Genetics of Hamad Medical Corporation. BDS is an employee of GeneDx, an Opko Health Company, and has stock options in Opko. GeneDx and HMC and the authors work together in multiple clinical and research areas. BDS is the Deputy Editor‐in‐Chief of American Journal of Medical Genetics, part A. The authors declare that there are no other conflicts of interest.
